# Interfollicular communication among preovulatory follicles after luteinizing hormone signaling

**DOI:** 10.1210/endocr/bqag105

**Published:** 2026-09-16

**Authors:** Corie M Owen, Siu-Pok Yee, Laurinda A Jaffe

**Affiliations:** Department of Cell Biology, University of Connecticut Health Center, Farmington, CT 06030, USA; Department of Cell Biology, University of Connecticut Health Center, Farmington, CT 06030, USA; Center for Mouse Genome Modification, University of Connecticut Health Center, Farmington, CT 06030, USA; Department of Cell Biology, University of Connecticut Health Center, Farmington, CT 06030, USA

**Keywords:** luteinizing hormone, ovary, oocyte meiosis, mouse, epidermal growth factor receptor, interfollicular communication

## Abstract

Luteinizing hormone (LH) triggers the resumption of oocyte meiosis and ovulation in preovulatory ovarian follicles. These events have generally been viewed as autonomous responses occurring independently within each follicle. Here, however, we show that mouse preovulatory follicles can communicate with one another through an LH-induced paracrine signaling network. Isolated preovulatory follicles lacking LH receptors (*Lhr*-KO) resumed oocyte meiosis when cocultured with LH-stimulated wildtype follicles, despite being unable to respond directly to LH. Oocytes within *Lhr*-KO follicles also resumed meiosis when exposed to conditioned medium from LH-treated wildtype follicles, demonstrating that diffusible factors mediate this interfollicular communication. Neutralizing antibodies against the epidermal growth factor receptor ligands epiregulin and amphiregulin inhibited the LH-induced interfollicular communication, identifying these LH-induced factors as key signaling molecules. Although epiregulin and amphiregulin are known to transmit LH signals within individual follicles, our findings indicate that they can also coordinate responses among neighboring follicles. Together, these results demonstrate that LH regulates a communication network between preovulatory follicles rather than acting solely at the level of individual follicles.

Mammalian oocytes form during embryonic development, during which time they enter meiosis (1). The chromosomes duplicate and undergo recombination, but then the meiotic cell cycle arrests, to resume only after puberty, in response to the cyclical release of luteinizing hormone (LH) from the pituitary. Each oocyte is located within a complex of somatic cells, comprising an ovarian follicle. As the follicle grows to the preovulatory stage, the granulosa and theca cells proliferate to form more than 10 cellular layers (Fig. 1A), and the outermost layer of granulosa cells synthesizes receptors for LH (3). LH elicits signals that travel inwards to the oocyte to cause meiosis to resume (4). LH also acts to cause structural changes in the follicle to release the oocyte at ovulation, such that fertilization can occur (5, 6).

**Figure 1:**
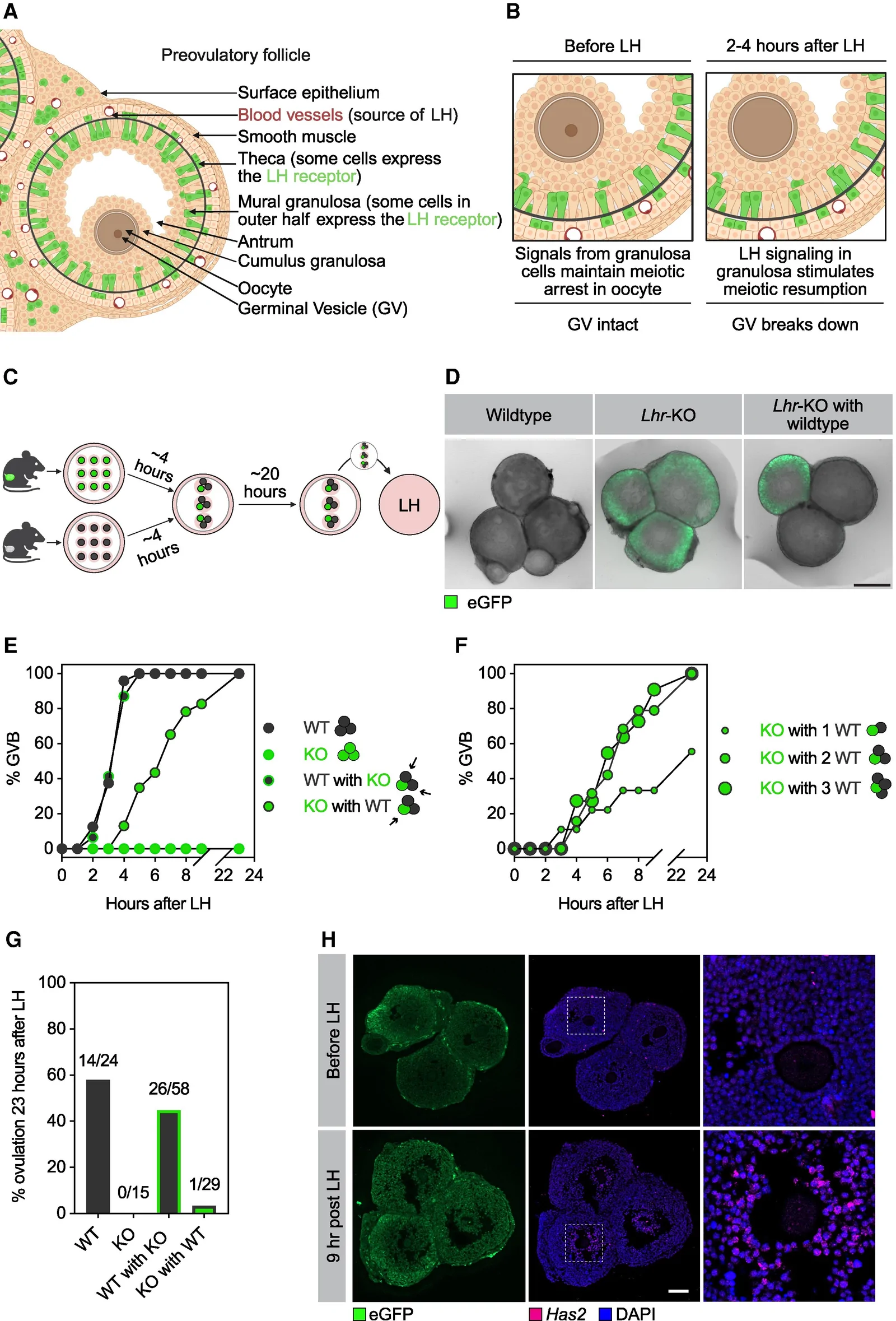
When cocultured with wildtype follicles, *Lhr*-KO follicles undergo LH-induced meiotic resumption and their cumulus cells transcribe hyaluronan synthase, a key step in the process leading to ovulation. (A) Structure of a mouse preovulatory follicle and localization of LH receptors. (B) Schematic of GVB, indicating resumption of meiosis in the follicle in response to LH signaling. (C) Preparation of cocultures. Large antral follicles from *Lhr*-KO and wildtype mice were placed on optically clear Millicell culture plates and then arranged in clusters and cultured overnight in the presence of FSH. The Millicell was then transferred to a dish with LH, and follicles were observed hourly for the occurrence of GVB. (A-C) were made in Biorender.com. (D) Follicle clusters showing eGFP fluorescence in the *Lhr*-KO follicles: 3 wildtype follicles, 3 *Lhr*-KO follicles, or 1 *Lhr*-KO with 2 wildtype follicles (scale bar = 250 µm). (E) Time course of LH-induced GVB in clusters of follicles as shown in (D). Arrows indicate the follicles in the cluster that the data represent. Data from 108 follicles. (F) Time course of LH-induced GVB in *Lhr*-KO follicles cocultured with different numbers of wildtype follicles. Data from 43 follicles including 23 also shown in (E). (G) LH-induced ovulation in clusters of follicles of the indicated genotypes. Data are from the follicles shown in (E), and from 6 time-lapse recordings like that shown in Video S1 (2). Numbers indicate the fraction of follicles that had ovulated at 23 hours after LH addition. (H) LH-induced transcription of mRNA for hyaluronan synthase (*Has2*) in wildtype and *Lhr*-KO follicles in cocultures. Follicle clusters were fixed and processed for cryosectioning, before or 9 hours after applying LH, a time point at which almost all oocytes in *Lhr*-KO follicles had undergone GVB. *Lhr*-KO follicles were identified by eGFP fluorescence in their mural granulosa cells (left panels); eGFP fluorescence was visible above the autofluorescence of the fixed tissue. *Has2* mRNA labeling was seen in the cumulus region only after LH (middle panels, with boxed regions at higher magnification in right panels). Scale bar = 100 µm.

Although LH can stimulate meiotic resumption (7, 8) and ovulation (5, 9, 10) in isolated preovulatory follicles in culture, there are clues that signals originating from LH receptors elsewhere in the ovary could also contribute to responses in an individual follicle. Only a small fraction of the total ovarian LH receptor protein is present in any individual mouse preovulatory follicle, and LH receptor expression among preovulatory follicles is highly variable (3, 11). The synchrony (11-13) of ovulation in polyovulatory species, in which multiple eggs are released into the oviduct ready for fertilization, and the LH-induced secretion of diffusible second messengers into the follicular extracellular space (14), suggest that LH signals are coordinated by a follicular network.

Previous studies have demonstrated that signaling between mouse ovarian follicles contributes to regulation of their growth (15-18). Analysis of ovarian sections has shown that primordial follicles that are close to other primordial follicles are less likely to begin growth (15), but that primordial follicles located within 40 μm of ovulating follicles are more likely to begin growth (16). Follicle coculture experiments have also provided evidence for interfollicular communication. A preantral follicle that is cultured in contact with another preantral follicle suppresses growth of its neighbor (17), and a preantral follicle that is cultured in contact with a neonatal ovary suppresses the initiation of growth in adjacent primordial follicles (18). Here, we investigate whether there is interfollicular communication between adjacent preovulatory follicles, during stimulation by LH. By coculturing isolated preovulatory follicles lacking LH receptors with wildtype preovulatory follicles, we show that the cocultured LH receptor-deficient follicles can resume meiosis in response to LH.

## Materials and methods

### Mice

The *Lhr*-KO mice, also known as *Lhr^INV/INV^*, have the coding sequence of the *Lhr* gene replaced with eGFP (19). The *Lhr*-KO mice were generated by heterozygous breeding pairs (*Lhr^INV^*^/+^*×Lhr*^INV/+^). The wildtype and knockout (INV) alleles were distinguished by PCR genotyping (19, 20). The background strain for these and the wildtype mice used here was C57BL/6J. All procedures were approved by the animal care committee of the University of Connecticut Health Center.

### Culture and imaging of follicles

Large antral follicles (290-360 µm in diameter) were dissected from 23- to 26-day old mice with no prior exogenous hormone treatment. The follicles were placed on 30 mm Millicell culture plates (PICMORG50, MilliporeSigma) as previously described (21) in medium (9) containing 1 nM ovine follicle stimulating hormone (FSH) (National Hormone and Peptide Program, AFP7558C; currently available from Golden West Companies, Temecula, CA).

For coculture experiments, the isolated follicles were cultured for ∼4 hours, and then rearranged to form clusters. For each cluster, 2-4 follicles were placed close together on the Millicell, then excess medium was removed from the drops using a mouth pipette (22), forcing the follicles closer together. Three to four clusters were cultured on each Millicell. After ∼20 hours, the Millicell was transferred to a glass coverslip bottom dish (Mattek #P35GINV-0-20-C) for quick fluorescence imaging on a Zeiss 980 confocal system on an inverted microscope to identify the *Lhr*-KO follicles based on eGFP fluorescence. The Millicell was then moved into a 35 mm dish with 2 mL of culture medium with 10 nM LH (National Hormone and Peptide Program, ovine LH-26; currently available from Golden West Companies). Meiotic resumption was assessed every hour by checking for the breakdown of the prophase arrested nucleus known as the germinal vesicle (GVB) (Fig. 1B), using an upright microscope with a 20×/0.4 NA/∼11 mm working distance LD ACHROPLAN objective (Zeiss #44 08 44). Time-lapse imaging of follicle clusters was performed as previously described (19).

To test the effect of epidermal growth factor receptor (EGFR) ligands, isolated follicles were cultured on Millicells for 23-25 hours, then the Millicells were moved into dishes containing 2 mL medium with mouse epiregulin (Bio-techne R+D Systems, #1068-EP, 100 nM) and mouse amphiregulin (Peprotech #315-36, 100 nM).

To test the effect of EGFR kinase inhibition on responses to LH, follicle clusters were preincubated for 1 hour in 200 nM AG1478 (Sigma, #658552) or in control medium containing 0.1% dimethylsulfoxide (DMSO), and then LH was added. To test the effect of neutralizing antibodies targeting EGFR ligands, follicle clusters were preincubated for 1 hour with a mixture of 100 µg/mL (∼700 nM) of a rat monoclonal antibody against mouse epiregulin (R&D Systems MAB1068, clone #189611, RRID: AB_2098304) (23) and 100 µg/mL (∼700 nM) of a mouse monoclonal antibody against mouse/human amphiregulin (Bio X Cell BE0454, clone #AR37, RRID: AB_3696177) (24), or with a mixture of control antibodies (100 µg/mL rat IgG2A, R&D Systems MAB006, clone #54447, R(2)RID: AB_357349, and 100 µg/mL mouse IgG, Bio X Cell BE0093, RRID: AB_1107789). LH was then added to the dishes.

### Detection of LH-induced transcription of hyaluronan synthase in follicle clusters

Clusters of 2 wildtype follicles and 1 *Lhr*-KO follicle were fixed in 4% paraformaldehyde in PBS, before or after a 9-hour exposure to 10 nM LH. The fixed clusters were processed and cryosectioned as previously described (3). Sections were imaged using an LSM 980 confocal microscope to identify the *Lhr*-KO follicle based on eGFP fluorescence. HCR Gold RNA-FISH (25) was then performed using a mouse *Has2* X2 probe (Molecular Instruments) with incubation at 37 °C in a humidified chamber overnight, and hairpin X2 647 (Molecular Instruments) with incubation for 3 hours at room temperature in the dark. Sections were labeled with DAPI (Sigma #D9542) to mark cell nuclei, and then reimaged using an LSM 980 confocal microscope, with a 10×/0.5 NA Plan-Apochromat objective. Z-stack images were collected at 1 µm intervals over a 10 µm range, and then displayed as a maximum projection. For clarity, brightness, and contrast were adjusted using FIJI software (26), with the same settings for images of samples with and without LH.

### Experiments with conditioned medium

For experiments with conditioned medium, follicles from wildtype mice were dissected and placed on Millicells, and 4 hours later follicles from *Lhr*-KO mice were dissected and cultured similarly. 24 hours after dissection, 2-12 wildtype follicles were placed on a ∼4 × 4 mm raft of Millicell membrane, located on top of a Millicell culture plate. The raft was then moved to the top of a 30 µL drop of medium with 10 nM LH in a 16-well microwell plate (IVF Store, BIRR 16-well dish, #113016). A similar raft of follicles was placed on top of a control 30 µL drop of medium without LH. Another control consisted of a raft without follicles placed on top of a 30 µL drop of medium with 10 nM LH. Wet Kimwipes were placed in a “moat” surrounding the 16 wells to maintain high humidity. After 4 or 8 hours, the 30 µL of medium under each Millicell raft was transferred to another microwell, and the rafts with wildtype follicles were returned to the surface of a Millicell culture plate for observation. A Millicell raft with 4 *Lhr*-KO follicles was then transferred to the 30 µL drop of conditioned medium or to the control drops. 4 hours later, the Millicell rafts with *Lhr*-KO follicles were returned to a Millicell culture plate for observation of GVB.

For some experiments, 30 µL aliquots of conditioned medium were frozen in liquid nitrogen and stored at −80 °C. For documenting the presence of a polar body and the appearance of the cumulus cells, follicles were opened using 30-gauge needles, and cumulus–oocyte complexes were photographed using an upright microscope with a 20×/0.4 NA/∼11 mm working distance LD ACHROPLAN objective and an iPhone 13 Pro camera attached with a LabCam microscope adapter (iDu Optics).

### Detection of LH-induced transcription of epiregulin in mouse ovaries

The 22-24 day-old wildtype mice were injected with 5 I.U. equine chorionic gonadotropin (ProSpec #HOR-272) to mimic the action of FSH and cause follicles to develop to the preovulatory stage (27); 44 hours later, the mice were injected with 1 nmol kisspeptin (Cayman Chemicals, #24477) to stimulate release of endogenous LH (12). Ovaries were fixed before or 2 hours after kisspeptin injection, frozen, and sectioned (3). HCR Gold RNA-FISH (25) and imaging were performed as described above, using a mouse *Ereg* X2 probe and hairpin X2 488.

## Results

### *Lhr*-KO follicles undergo LH-induced meiotic resumption when cocultured with wildtype follicles

For coculture of follicles with and without LH receptors, we used an LH receptor knockout mouse line in which LH receptors were replaced by eGFP (19). Preovulatory follicles (Fig. 1A) were obtained by dissecting fully grown follicles from ∼24-day old mice and culturing them for ∼24 hours with FSH to activate the LH receptor promoter (see Materials and Methods). In response to LH, oocytes within wildtype follicles resumed meiosis, as indicated by breakdown of the GVB (Fig. 1B). As previously described, isolated *Lhr*-KO follicles did not undergo GVB in response to LH (19).

Follicle cocultures were prepared as outlined in Fig. 1C. The *Lhr*-KO follicles, which had been identified by genotyping, could be visually distinguished from the wildtype follicles within a coculture by their green fluorescence (Fig. 1D). As expected, wildtype follicles in these clusters resumed meiosis in response to LH, while *Lhr*-KO follicles in clusters with other *Lhr*-KO follicles did not (Fig. 1E). Remarkably, however, *Lhr*-KO follicles in clusters with wildtype follicles underwent GVB in response to LH (Fig. 1E). GVB in the *Lhr*-KO follicles occurred with a ∼2-4-hour delay compared with wildtype follicles (Fig. 1E). Coculture with a single wildtype follicle was sufficient to allow an *Lhr*-KO follicle to resume meiosis, although the fraction of follicles responding was smaller than in cultures with 2 or 3 wildtype follicles (Fig. 1F).

### *Lhr-*KO follicles cocultured with wildtype follicles do not ovulate in response to LH, but their cumulus cells transcribe hyaluronan synthase, a key step in the process leading to cumulus expansion and ovulation

In addition to restarting meiosis, LH signaling causes ovulation (5, 6). While about half of the wildtype follicles in cocultures ovulated in response to LH, *Lhr*-KO follicles in cocultures with the wildtype follicles did not (Fig. 1G; Video S1) (2). Although oocytes were not released from these follicles, we tested for the occurrence of one of the essential steps in the sequence of LH-induced events that leads to oocyte release, transcription of mRNA for hyaluronan synthase (HAS2) (28). HAS2 synthesizes hyaluronic acid, which together with follicular proteins forms an extracellular matrix between the cumulus cells, causing the cumulus cells to disperse from the oocyte surface and from each other in a process called cumulus expansion (8, 29-31). The formation of the extracellular matrix draws fluid into the antral space, causing the follicular volume to increase, which is necessary for ovulation (5).

*Has2* mRNA was seen in the cumulus region of 5/6 *Lhr*-KO follicles that were cocultured with wildtype follicles and fixed at 9 hours after LH application (Fig. 1H). 0/5 *Lhr*-KO follicles in cocultures without LH showed *Has2* mRNA in the cumulus (Fig. 1H). These results showed that although *Lhr*-KO follicles cocultured with wildtype follicles do not ovulate in response to LH, their cumulus cells transcribe hyaluronan synthase, a key step in the process leading to cumulus expansion and ovulation.

### Interfollicular communication is mediated by a soluble factor

To investigate if follicular contact was required for interfollicular communication, we tested whether culture medium conditioned by incubation of wildtype follicles in the presence of LH would cause meiotic resumption in *Lhr*-KO follicles. To generate a concentrated solution, we used a microwell procedure as outlined in Fig. 2A. The conditioned medium stimulated GVB in *Lhr*-KO follicle-enclosed oocytes (Fig. 2B). Fewer wildtype follicles, or a shorter conditioning period, generated less activity (Fig. 2B). Control medium conditioned with wildtype follicles, but without LH, lacked activity, as did medium with LH but without wildtype follicles (Fig. 2B).

**Figure 2:**
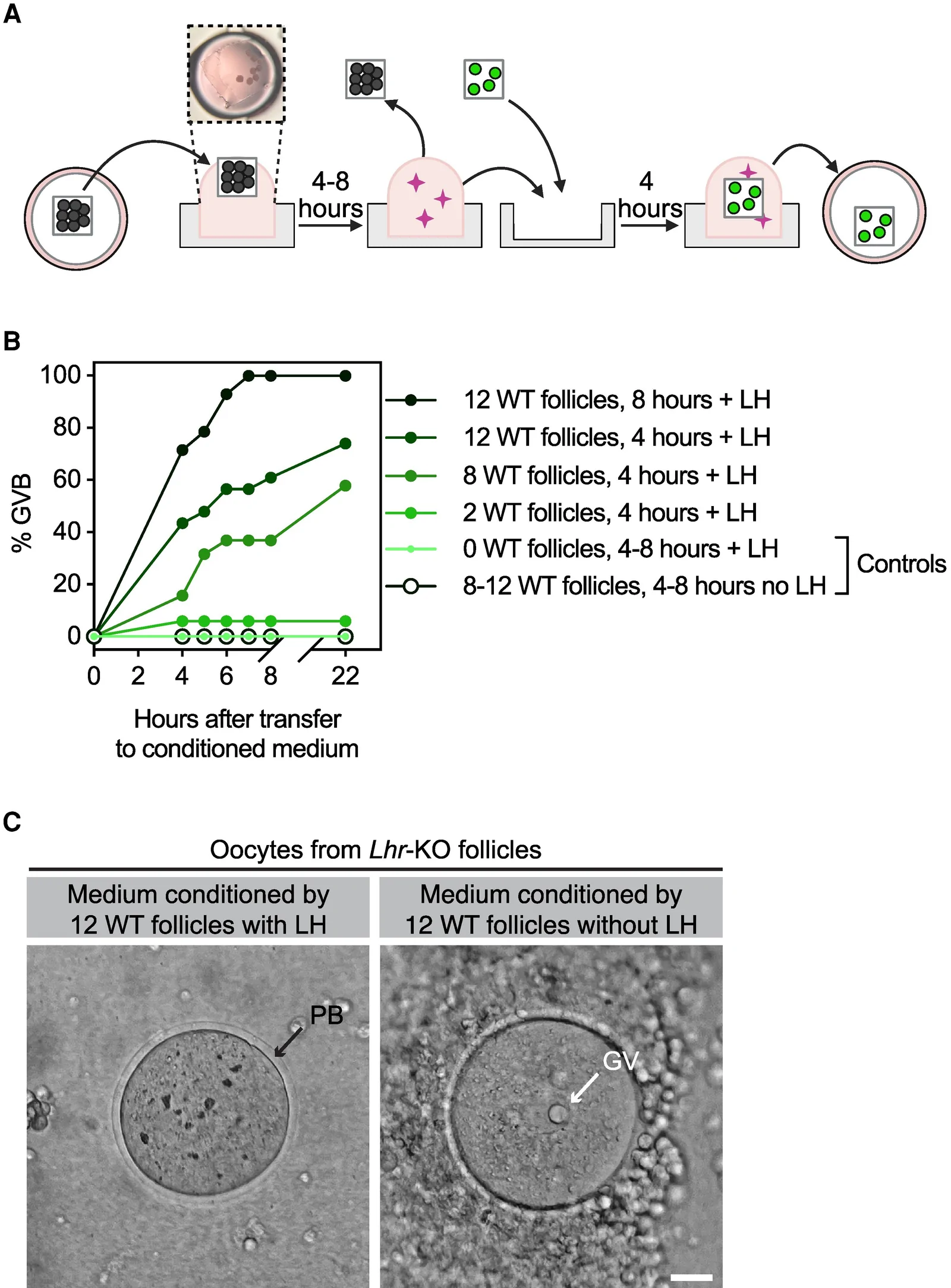
Interfollicular communication is mediated by a soluble factor. (A) Procedure for generating and testing conditioned medium. Wildtype follicles (black) were cultured with LH on a 4 × 4 mm raft of Millicell membrane floated on top of a 30 µL drop of medium in a microwell plate. A photo of a raft of follicles on a microdrop is shown in the box marked by dashed lines. After 4-8 hours, the medium was recovered and transferred to a new microwell. A Millicell raft with *Lhr*-KO follicles (green) was placed on top of this drop for 4 hours, and then moved to a 30 mm Millicell for observation of GVB. Figure made in Biorender.com. (B) Percent GVB as a function of time after *Lhr-*KO follicles were placed in contact with medium conditioned by incubation with 0-12 wildtype (WT) follicles and LH, or control medium conditioned with follicles without LH. Graph shows GVB data from 107 *Lhr*-KO follicles. (C) Oocytes isolated at 24 hours after exposing *Lhr*-KO follicles to medium conditioned with 12 wildtype follicles with or without LH. Scale bar = 25 µm. Note that oocyte diameters are increased due to flattening on the Millicell membrane.

Although the conditioned medium induced GVB in *Lhr*-KO follicles, it did not cause ovulation (0/33 follicles). However, at 24 hours after exposure to conditioned medium, the cumulus cells around the oocyte had dispersed, which occurs as a result of formation of the hyaluronic acid containing matrix between the cumulus cells. This could be seen through the follicular wall, and was documented by opening the follicle to release the oocyte (Fig. 2C, representative of 9/10 oocytes). Oocytes within these follicles had formed a polar body, indicating that meiosis had progressed to metaphase II (Fig. 2C, representative of 7/10 oocytes). In control *Lhr*-KO follicles that had been exposed to medium conditioned by wildtype follicles without LH, the cumulus cells remained tightly associated with the oocytes, and the oocytes remained arrested in prophase (Fig. 2C, representative of 6/6 oocytes).

### Interfollicular communication is mediated by EGFR ligands

LH stimulates meiotic resumption in follicle-enclosed oocytes by 2 parallel pathways, one of which regulates levels of cyclic nucleotides that diffuse through gap junctions between granulosa cells and the oocyte (4). The other pathway involves LH-induced synthesis of EGFR ligands, including epiregulin and amphiregulin, throughout the ovary (14, 32-34); 2 hours after kisspeptin injection of a mouse to induce release of endogenous LH (12), epiregulin mRNA was detected in granulosa cells of preovulatory follicles, and in theca and interstitial cells (Fig. 3A). EGFR activation causes phosphorylation and closure of gap junctions between granulosa cells (32, 33), which contributes to causing GVB (34). Thus, one way that LH signaling in wildtype follicles could stimulate GVB in an adjacent *Lhr-*KO follicle is by providing epiregulin and amphiregulin. As in wildtype follicles (14, 32), oocytes within isolated *Lhr*-KO follicles resumed meiosis in response to epiregulin and amphiregulin (Fig. 3B). The EGFR ligands did not cause ovulation in follicles of either genotype (Fig. 3B).

**Figure 3:**
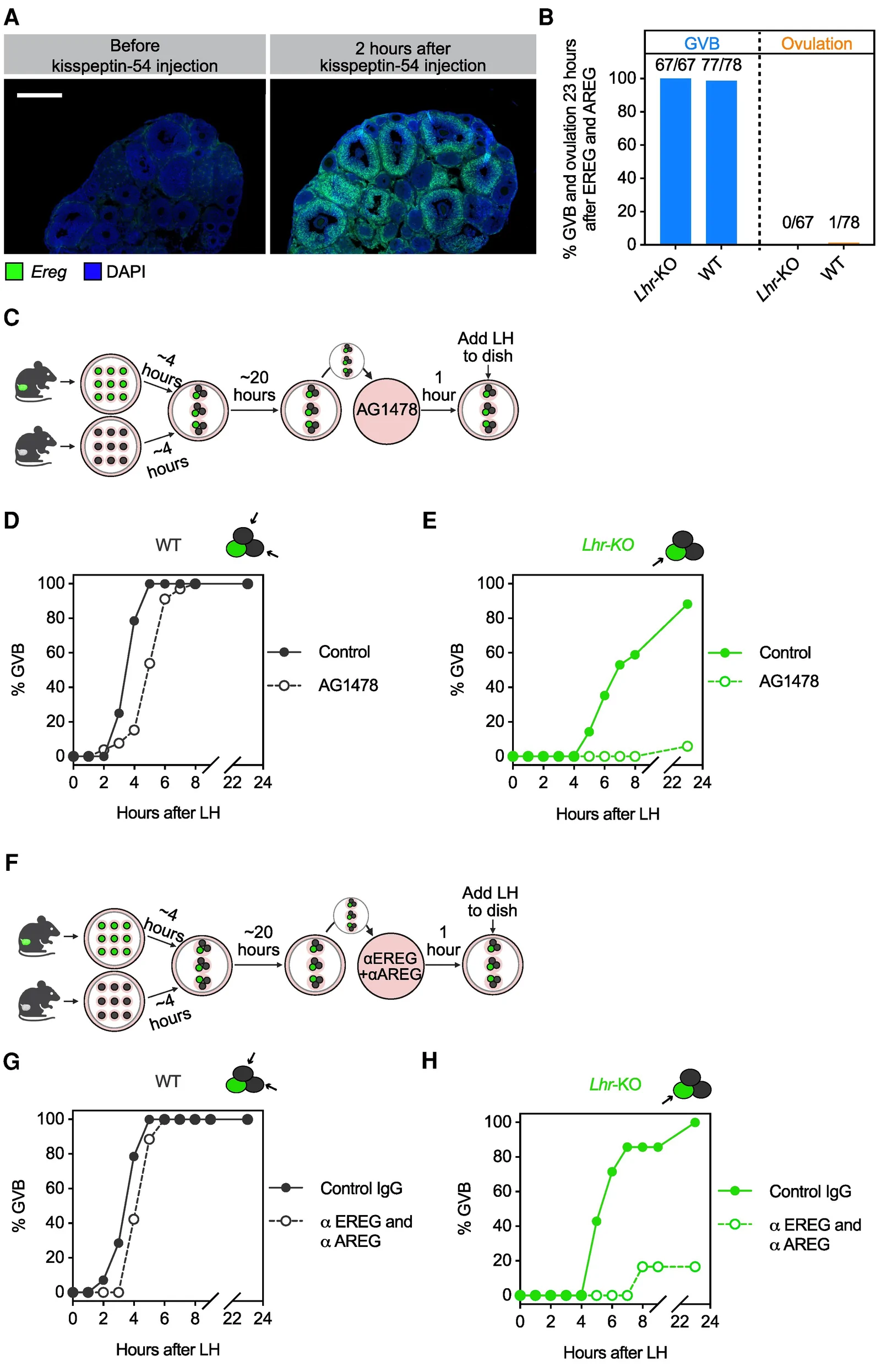
Interfollicular communication is mediated by EGFR ligands. (A) Epiregulin mRNA synthesis at 2 hours after injection of a wildtype mouse with kisspeptin to stimulate endogenous release of LH (12). Scale bar = 500 µm. (B) Percent GVB and ovulation in isolated wildtype and *Lhr*-KO follicles exposed to a mixture of 100 nM epiregulin (EREG) and 100 nM amphiregulin (AREG). Numbers of follicles are indicated above each bar. (C) Protocol for testing the effect of the EGFR kinase inhibitor AG1478 (200 nM) on LH-induced GVB in oocytes within clusters of *Lhr*-KO and wildtype follicles. (D) Time course of LH-induced GVB in the wildtype follicles in clusters as shown in (C). The control solution contained 0.1% DMSO. (E) Time course of LH-induced GVB in the *Lhr*-KO follicles in clusters as shown in (C). (D and E) show results from 102 follicles. (F) Protocol for testing the effect of neutralizing antibodies targeting epiregulin and amphiregulin (700 nM of each) on LH-induced GVB in oocytes within clusters of *Lhr*-KO and wildtype follicles. (G) Time course of LH-induced GVB in the wildtype follicles in clusters as shown in (F). (H) Time course of LH-induced GVB in the *Lhr*-KO follicles in clusters as shown in (F). (G and H) show results from 81 follicles. Diagrams were generated in Biorender.com.

To test whether EGFR ligands could be a paracrine factor mediating LH-induced meiotic resumption in *Lhr*-KO follicles cocultured with wildtype follicles, we incubated the cocultured follicles with the EGFR-selective kinase inhibitor AG1478 (200 nM) and then applied LH (Fig. 3C). In medium with AG1478, wildtype follicles in cocultures with an *Lhr*-KO follicle resumed meiosis in response to LH, although with a small delay (Fig. 3D). However, the *Lhr*-KO follicles in these cocultures with AG1478 did not respond to LH (Fig. 3E), supporting the hypothesis that EGFR ligands mediate the interfollicular communication.

We further tested this hypothesis by applying a mixture of amphiregulin and epiregulin-neutralizing antibodies (23, 24) to cocultures of wildtype and *Lhr*-KO follicles (Fig. 3F). The neutralizing antibodies slightly delayed LH-induced GVB in the wildtype follicles (Fig. 3G), and almost completely inhibited LH-induced GVB in the *Lhr*-KO follicles in these cocultures (Fig. 3H). These results indicate that the EGFR ligands that are synthesized in response to LH can convey signals between preovulatory follicles (Fig. 4).

**Figure 4:**
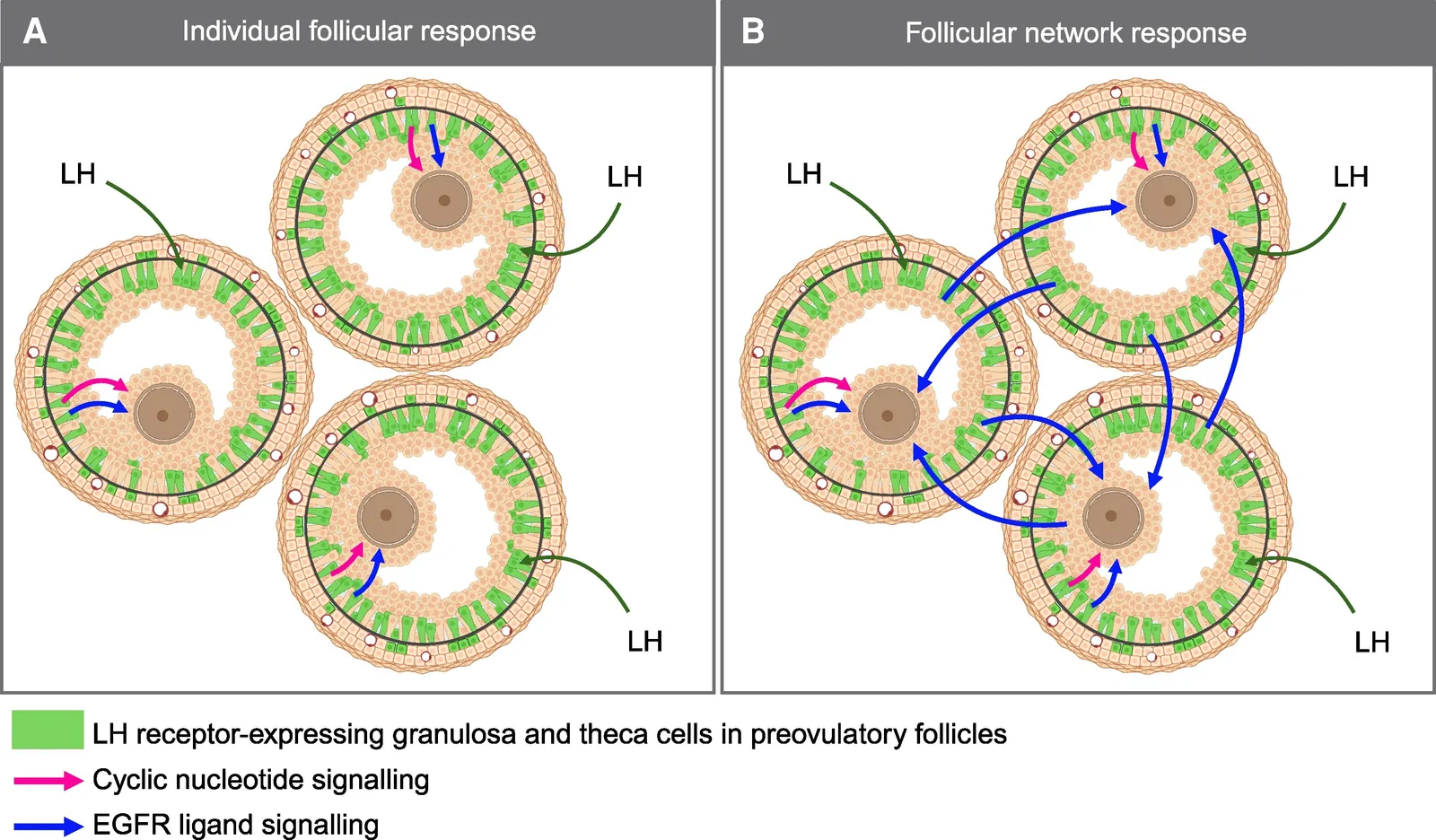
LH-induced interfollicular signaling. (A) LH induces signals within an individual preovulatory follicle, conveyed by cyclic nucleotides (pink arrows) and EGFR ligands (blue arrows). (B) LH also induces signals that travel between preovulatory follicles, conveyed by EGFR ligands (blue arrows). See Fig. 1A for labels of the cell types in a follicle. Figure made in Biorender.com.

## Discussion

Our results indicate that LH triggers an interfollicular response, in which EGFR ligands diffuse beyond individual follicle borders. Our studies were conducted with follicles placed together in clusters in vitro, raising the question of whether similar interfollicular communication also occurs in vivo. Preovulatory follicles in ovaries of adult mice (11, 16, 35, 36), rats (37) and hamsters (38) make contacts with each other that are similar to those in our in vitro cocultures, suggesting that EGF receptor ligands and other possible paracrine factors between adjacent follicles in vivo could be present at concentrations comparable to those attained in the spaces between follicles placed together in vitro. Thus, the interfollicular communication that occurs between follicles placed together in vitro could also occur in vivo, although this is unknown.

Our results also indicate that EGFR ligands released by the granulosa cells of preovulatory follicles in response to LH are a key component of the signal that travels to adjacent preovulatory follicles. Genetic modification of mice to partially reduce EGFR protein in the granulosa cells impairs ovulation and reduces the number of pups born per litter (33). Our findings suggest that the requirement of EGFR signaling for optimal reproduction is due not only to communication within follicles, but also between follicles. In polyovulatory species, this communication between follicles could contribute to synchronizing ovulation, ensuring that all eggs are available for fertilization simultaneously.

Although the EGFR ligands that are released from LH-treated follicles can elicit GVB, as well as the expansion of the cumulus mass that occurs due to synthesis and secretion of hyaluronic acid (14), EGFR ligands and other molecules that are released extracellularly in response to LH are not sufficient by themselves to cause ovulation. Thus, additional signaling events that occur within the cells of an individual follicle are evidently needed to cause all of the structural changes in the follicle that result in ovulation. These structural changes include thinning of the apical layers of mural granulosa cells, theca cells, and surface epithelium, followed by the transient opening of a hole through which the cumulus–oocyte complex is released (5, 6, 13, 39). The mechanisms responsible for apical thinning are poorly understood, but possible factors are the dissociation of mural granulosa cells due to secretion of extracellular matrix between the cells, proteolysis of the extracellular matrix, stretching of the apical surface due to expansion of the follicular volume, and migration of cells out of the apical region (6). Contraction of the smooth muscle in the theca layer in the basolateral region, mediated by endothelin-stimulated actomyosin contraction, is thought to cause rupture of the thinned follicular apex (5, 6). If the LH-induced signaling pathways that lead to apical thinning and basolateral contraction are mediated by molecules that are not released into the extracellular space, *Lhr*-KO follicles in coculture with wildtype follicles would not receive the signals.

EGFR ligands or other paracrine factors released in the ovary in response to the mid-cycle LH surge might also mediate interfollicular communication from preovulatory follicles to smaller follicles. EGFR levels increase as follicles approach the preovulatory stage (40, 41), but EGFR expression has been detected in follicles as early as the preantral stage in mice (42) and other mammalian species (40, 43-45), based on the presence of mRNA or protein, or the occurrence of cellular responses to EGF. EGF has little or no effect on growth of preantral mouse follicles cultured in vitro (42), but EGF treatment of isolated ovaries from 6-day old mice has been reported to increase the fraction of activated primordial follicles (46). Thus, the follicular network activated by the release of LH could act not only to coordinate preovulatory processes, but also to regulate the development of subsequent cohorts of developing follicles.

## Data Availability

The genetically modified mouse line used for this study has been deposited at the Mutant Mouse Resource and Research Center at The Jackson Laboratory (Bar Harbor, ME) (MMRRC #076250, C57BL/6J-*Lhcgr^em2Laj^*/Mmjax, JAX stock #041561). Datasets generated by this study are available by request to the corresponding authors. The video (S1) and additional similar videos can be found in the FigShare Repository: https://figshare.com/articles/figure/_/32736009.
